# Retraction Note: MicroRNA-338-3p suppresses ovarian cancer cells growth and metastasis: implication of Wnt/catenin beta and MEK/ ERK signaling pathways

**DOI:** 10.1186/s13046-024-02978-0

**Published:** 2024-02-15

**Authors:** Ruitao Zhang, Huirong Shi, Fang Ren, Wei Feng, Yuan Cao, Gailing Li, Zheying Liu, Pengcheng Ji, Minghui Zhang

**Affiliations:** Department of Gynecology, First Affiliated Hospital, Zhengzhou University, NO.1 East Jianshe Road, Erqi District, Zhengzhou, 450052 Henan People’s Republic of China

**Retraction Note**: ***J Exp Clin Cancer Res *38, 494 (2019)**

10.1186/s13046-019-1494-3

The Editor-in-Chief has retracted this article after concerns were raised about some of the data reported. Specifically:

- In Fig. 3B, the Con and Met + miR-338-3p images for OVCAR3 cells appear to be duplicates.
- In Fig. 4A, there appears to be partial overlap between the images of MACC1 and Met conditions and Con and Met + miR-338-3p conditions in OVCAR3 cells.
- In Fig. 4B, there appears to be partial overlap between the images of miR-338-3p and Met + miR-338-3p conditions of OVCAR3 cells.

The Editor-in-Chief no longer has confidence in the article’s results and conclusions.

Ruitao Zhang agrees with this retraction. The remaining authors did not respond to correspondence from the publisher about this retraction.

